# VGLUT3 Ablation Differentially Modulates Glutamate Receptor Densities in Mouse Brain

**DOI:** 10.1523/ENEURO.0041-22.2022

**Published:** 2022-05-09

**Authors:** Karim S. Ibrahim, Salah El Mestikawy, Khaled S. Abd-Elrahman, Stephen S.G. Ferguson

**Affiliations:** 1 Brain and Mind Research Institute, University of Ottawa, Ottawa, Ontario K1H 8M5, Canada; 2 Department of Cellular and Molecular Medicine, University of Ottawa, Ottawa, Ontario K1H 8M5, Canada; 3 Department of Pharmacology and Toxicology, Faculty of Pharmacy, Alexandria University, Alexandria 21521, Egypt; 4 Neuroscience Paris Seine–Institut de Biologie Paris Seine (NPS–IBPS) INSERM, CNRS, Sorbonne Université, 75006 Paris, France; 5 Department of Psychiatry, Douglas Hospital Research Centre, McGill University, Montreal, Quebec H4H 1R3, Canada

**Keywords:** brain, glutamate, GPCR, synaptic plasticity, transporter

## Abstract

Type 3 vesicular glutamate transporter (VGLUT3) represents a unique modulator of glutamate release from both nonglutamatergic and glutamatergic varicosities within the brain. Despite its limited abundance, VGLUT3 is vital for the regulation of glutamate signaling and, therefore, modulates the activity of various brain microcircuits. However, little is known about how glutamate receptors are regulated by VGLUT3 across different brain regions. Here, we used VGLUT3 constitutive knock-out (VGLUT3^–/–^) mice and explored how VGLUT3 deletion influences total and cell surface expression of different ionotropic and metabotropic glutamate receptors. VGLUT3 deletion upregulated the overall expression of metabotropic glutamate receptors mGluR5 and mGluR2/3 in the cerebral cortex. In contrast, no change in the total expression of ionotropic NMDAR glutamate receptors were observed in the cerebral cortex of VGLUT3^–/–^ mice. We noted significant reduction in cell surface levels of mGluR5, NMDAR2A, NMDAR2B, as well as reductions in dopaminergic D_1_ receptors and muscarinic M1 acetylcholine receptors in the hippocampus of VGLUT3^–/–^ mice. Furthermore, mGluR2/3 total expression and mGluR5 cell surface levels were elevated in the striatum of VGLUT3^–/–^ mice. Last, AMPAR subunit GluA1 was significantly upregulated throughout cortical, hippocampal, and striatal brain regions of VGLUT3^–/–^ mice. Together, these findings complement and further support the evidence that VGLUT3 dynamically regulates glutamate receptor densities in several brain regions. These results suggest that VGLUT3 may play an intricate role in shaping glutamatergic signaling and plasticity in several brain areas.

## Significance Statement

Type 3 vesicular glutamate transporter (VGLUT3) is an atypical vesicular glutamate transporter that is discretely expressed by subpopulations of nonglutamatergic neurons within the brain. Through these neurons, VGLUT3 regulates mood, movement coordination, rewarding behavior, and cognition. Despite extensive research on VGLUT3 neurotransmission in nonglutamatergic neurons, little is known on how glutamate receptors are regulated by VGLUT3 signaling in the brain. Using VGLUT3 knockout in mice, we show that VGLUT3 differentially regulates glutamate receptor densities in various brain regions, further supporting its critical role in regulating synaptic function and plasticity in brain circuits.

## Introduction

The atypical type 3 vesicular glutamate transporter (VGLUT3) is a unique member of the Solute Carrier 17a transporter family that mediates vesicular packaging of glutamate in primarily nonglutamatergic varicosities, as well as in subpopulations of glutamatergic neurons in the brain (Fremeau et al., 2004; El Mestikawy et al., 2011). VGLUT3 is expressed by discrete populations of neurons and interneurons in the cerebral cortex, raphe nuclei, hippocampus, striatum, olfactory bulb, and inner hair cells, and is transiently expressed in the cerebellum (Gras et al., 2002; Herzog et al., 2004; El Mestikawy et al., 2011). In addition to its well characterized vesicular glutamate transport functions, VGLUT3 modulates acetylcholine, serotonin, and GABA transmission (Gras et al., 2002; Somogyi et al., 2004; Trudeau and El Mestikawy, 2018). Functionally, VGLUT3 plays an important role in the neuronal networks involved in motor coordination, reward processing, and cognition (Ibrahim et al., 2020).

Evidence shows that VGLUT3 can modify glutamate release and mediate tonic excitatory postsynaptic inputs via both ionotropic glutamate receptor (iGluR) and metabotropic GluR (mGluR; Nelson et al., 2014; Sakae et al., 2015; Fasano et al., 2017). For instance, genetic deletion of VGLUT3 diminishes excitatory currents from tonically active interneurons that is mediated via AMPARs and NMDARs on the medium spiny neurons and GABAergic interneurons of the striatum (Higley et al., 2011; Nelson et al., 2014). Similarly, VGLUT3 regulates dopamine and GABA efflux via mGluR-dependent mechanisms in the nucleus accumbens and in the hippocampus (Sakae et al., 2015; Fasano et al., 2017). The overlapping distribution of VGLUT3 with different members of mGluRs strongly suggests that VGLUT3/mGluR cross talk is vital for synaptic plasticity modulation in various brain microcircuits (Fasano et al., 2017; Ibrahim et al., 2020). However, it remains unclear how GluRs can be regulated by VGLUT3 neurotransmission across different brain regions. In this study, we used VGLUT3 knock-out mice (VGLUT3^−/−^) to assess whether the loss of VGLUT3 alters total and cell surface expression of GluRs in the brain.

We report that the loss of VGLUT3 in mice upregulates the total expression of mGluR5 and mGluR2/3, while it downregulates NMDA2B and dopaminergic D_1_ (D1R) cell surface expression in the cerebral cortex. Furthermore, significant reductions in cell surface expression of mGluR5, NMDAR2A/B, D1R, and muscarinic M1 acetylcholine receptor (M1 mAChR) are observed in the hippocampus of VGLUT3^−/−^ mice. In addition, mGluR2/3 total expression and mGluR5 cell surface levels are higher in the striatum of VGLUT3^–/–^ mice. Finally, AMPAR subunit GluA1 total expression is upregulated throughout cortical, hippocampal, and striatal brain regions of VGLUT3^−/−^ mice. Together, these results provide evidence that GluR densities are dynamically regulated by VGLUT3 in several brain areas, highlighting the key role of this atypical transporter in the regulation of glutamatergic signaling, and potentially synaptic plasticity in these circuits.

## Materials and Methods

### Reagents

NeutrAvidin agarose beads (catalog #29200), goat anti-rabbit (catalog #G-21234) IgG (H+L) cross-adsorbed horseradish peroxidase secondary antibody, and rabbit anti-β-actin (catalog #PA1-183) were from Thermo Fisher Scientific. Rabbit anti-mGluR 2/3 (catalog #ab6438) and anti-vinculin (catalog #ab129002) were from Abcam. Rabbit anti-mGluR5 (catalog #AB5675), anti-NMDAR2A (catalog #AB1555), anti-NMDAR2B (catalog #AB1557), anti-AMPA1 (catalog #AB1504), and anti-M1 mAChR (catalog #M9808) were from Sigma-Aldrich. Reagents used for Western blotting were from BIO-RAD, and all other biochemical reagents were from Sigma-Aldrich.

### Animals

All experimental protocols were approved by the Institutional Animal Care Committee and in compliance with the Canadian Council of Animal Care guidelines. Animals were group housed under a constant 12 h light/dark cycle and given food and water *ad libitum*. Heterozygous knock-out VGLUT3 mice (VGLUT3^–/+^) were crossed with C57BL/6 mice to establish a mouse colony. Six-month-old wild-type (WT) and homozygous VGLUT3^−/−^ male littermates were used for experiments.

### Cell surface biotinylation and immunoblotting

Cell surface biotinylation and immunoblotting experiments were performed as previously reported (Abd-Elrahman et al., 2018; Ibrahim et al., 2021). Mice were euthanized by live cervical dislocation followed by dissection of the cerebral cortex, hippocampus, and striatum. Tissues were cross-chopped with a McIlwain tissue chopper with a slice thickness of 300 μm and were recovered for 40 min at 37°C in ACSF solution (127 mm NaCl, 2 mm KCl, 1 mm CaCl_2_, 1 mm MgSO_4_, 10 mm glucose, 1.2 mm KH_2_PO_4_, 26 mm NaH_2_CO_3_, pH 7.4) gassed with 95% O_2_/5% CO_2_. Samples were transferred to tubes and incubated with 1 mg/ml sulfo-NHS-SS-biotin in ACSF for 45 min on ice, and then washed three times with ACSF. Biotinylation was then quenched with 100 mm glycine in ACSF for 70 min on ice. Following three washes in ACSF, samples were homogenized and lysed in RIPA buffer (1% Nonidet P-40 substitute, 0.5% sodium deoxycholate, 0.1% SDS, 50 mm Tris-HCl, 150 mm NaCl, and 5 mm EDTA, pH 7.2) containing protease inhibitors (100 μm 4-benzenesulfonyl fluoride hydrochloride, 80 nm aprotinin, 2 μm leupeptin, 5 μm bestatin, 1.5 μm E-64, and 1 μm pepstatin A). Protein lysates were centrifuged at 18,000 × *g* at 4°C for 10 min. The supernatant was then collected, and total protein concentrations were quantified using a DC Protein Assay Kit (BIO-RAD). Biotinylated fractions in 500 μg samples were captured with NeutrAvidin agarose beads and were eluted in β-mercaptoethanol containing 3× SDS sample buffer (187.5 mm Tris-HCl, 6% SDS, 30% glycerol, 0.006% Bromophenol Blue). Total protein lysates were also aliquoted and diluted to 1 μg/μl in a mixture of lysis buffer and β-mercaptoethanol containing 3× SDS sample buffer and boiled for 10 min at 90°C.

Aliquots containing a total of 30–40 μg of proteins were resolved by electrophoresis on 6% and 7.5% SDS-PAGE and transferred onto nitrocellulose membranes. Blots were blocked in Tris-buffered saline, pH 7.6, containing 0.05% Tween 20 (TBST) and 5% nonfat dry milk for 1 h at room temperature. Blots were then incubated overnight at 4°C with primary antibodies diluted (1:1000) in TBST containing 1% nonfat dry milk. Membranes were washed three times in TBST and incubated with secondary antibodies (anti-rabbit) diluted (1:5000) in TBST containing 1% nonfat dry milk for 1 h. Blots were imaged using the BIO-RAD Chemidoc Gel Imaging System. Band densities were quantified using Image Lab Software and normalized to the loading control.

### Statistical analysis

Mean ± SEM values and sample sizes for each independent experiment are shown in the figure legends. Data were analyzed with GraphPad Prism 9 for normality and statistical significance. A two-tailed unpaired Student’s *t* test was used for comparisons. The significance level was set at *p* < 0.05.

## Results

### VGLUT3 deletion differentially alters glutamatergic and nonglutamatergic receptor densities in the mouse cerebral cortex

**Figure 1.**
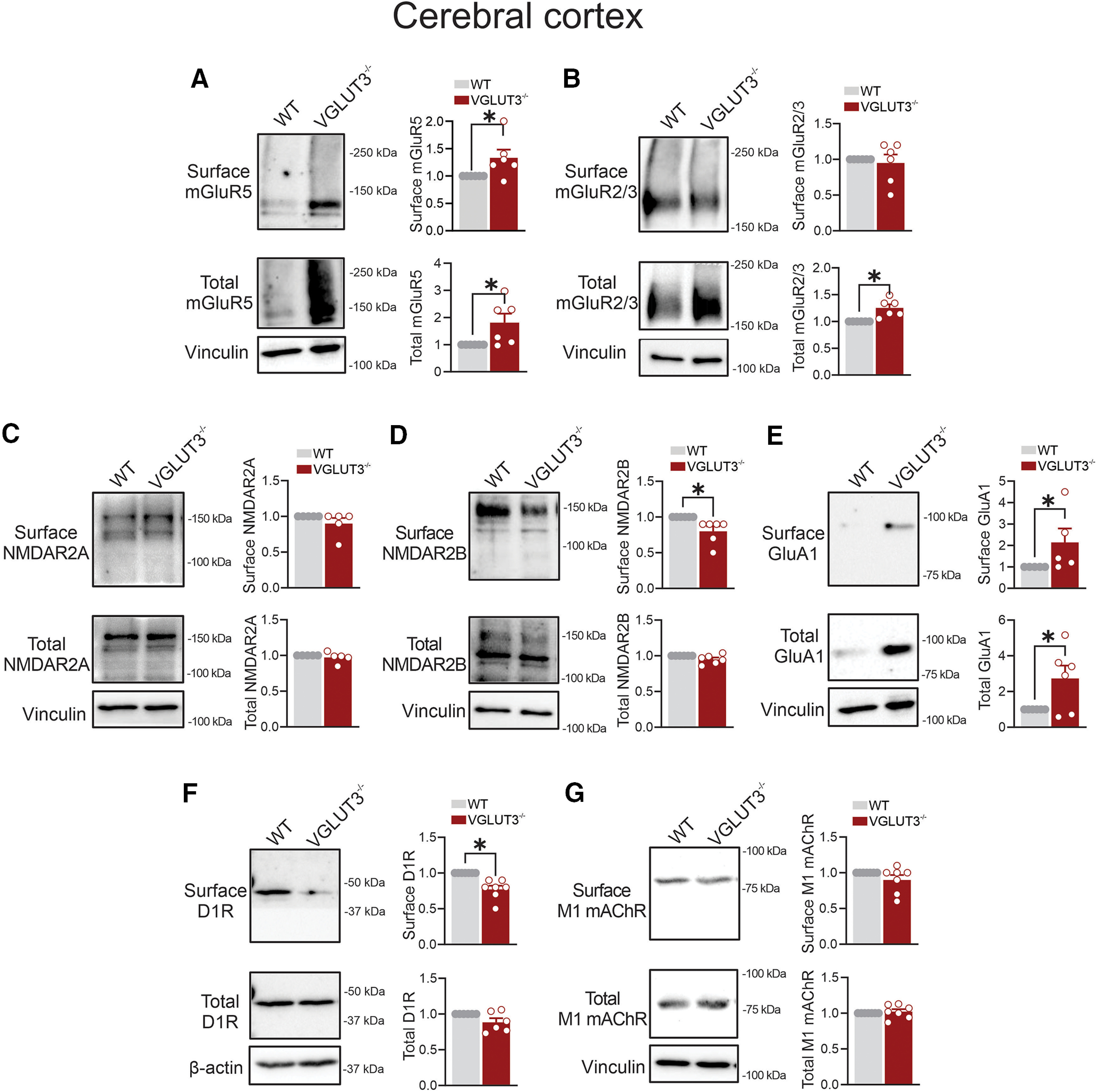
The effect of VGLUT3 deletion on glutamate and nonglutamate receptor expression in the mouse cerebral cortex. ***A–G***, Representative immunoblots and quantification of cell surface and total levels of mGluR5 (***A***), mGluR2/3 (***B***), NMDAR2A (***C***), NMDAR2B (***D***), GluA1 (***E***), D1R (***F***), and M1 mAChR (***G***) in cortical lysates from VGLUT3^−/−^ and C57BL/6 (WT) male mice. Data are the mean ± SEM (*n* = 5–6 mice/group), normalized to the respective surface or total WT values. *p < 0.05 assessed by unpaired Student’s *t* test.

VGLUT3 is localized in cortical layers II and VI as well as in subpopulations of interneurons within the cerebral cortex (Schäfer et al., 2002; Herzog et al., 2004). Therefore, we assessed the cell surface densities of mGluR5 and mGluR2/3, as they actively modulate synaptic plasticity responses in cortical circuits and are potentially regulated by VGLUT3-expressing fibers (Ballester-Rosado et al., 2010; Joffe et al., 2020; Abd-Elrahman and Ferguson, 2022). We found that VGLUT3 deletion significantly elevated mGluR5 and mGluR2/3 expression levels in the cerebral cortex of VGLUT3^−/−^ male mice compared with WT mice (Fig. 1*A*,*B*). Both NMDAR and AMPAR mediate VGLUT3 signaling in different brain areas (Higley et al., 2011; Nelson et al., 2014; Wang et al., 2019). We therefore assessed the expression of iGluR members NMDARs and AMPARs via probing for their essential regulatory subunits NMDAR2A/2B and GluA1, respectively (Paoletti et al., 2013; Henley and Wilkinson, 2016). We detected no change in the total expression levels of NMDAR2A/2B subunits in the cortex of VGLUT3^−/−^ mice compared with WT mice (Fig. 1*C*,*D*). However, cell surface expression of NMDAR2B, but not NMDAR2A, was significantly reduced in the cortex of VGLUT3^−/−^ mice (Fig. 1*C*,*D*). On the contrary, the loss of VGLUT3 significantly upregulated cell surface and total expression levels of AMPAR subunit GluA1 (Fig. 1*E*).

Given that VGLUT3^+^ neurons actively regulate dopaminergic and cholinergic neurotransmission (Trudeau and El Mestikawy, 2018), we probed for dopaminergic D1R and muscarinic M1 mAChR that are enriched in cortical GABAergic interneurons and pyramidal neurons, respectively (Levey et al., 1991; Anagnostaras et al., 2003; Puig et al., 2014). VGLUT3 ablation did not alter the total expression of either D1R or M1 mAChR in the cortex of VGLUT3^−/−^ mice (Fig. 1*F*,*G*). However, cell surface D1R level, but not M1 mAChR level, was significantly reduced in the cortex of VGLUT3^−/−^ mice (Fig. 1*F*,*G*).

### Deletion of VGLUT3 reduces cell surface levels of mGluR5, NMDAR, D1R, and M1 mAChR in the hippocampus

**Figure 2.**
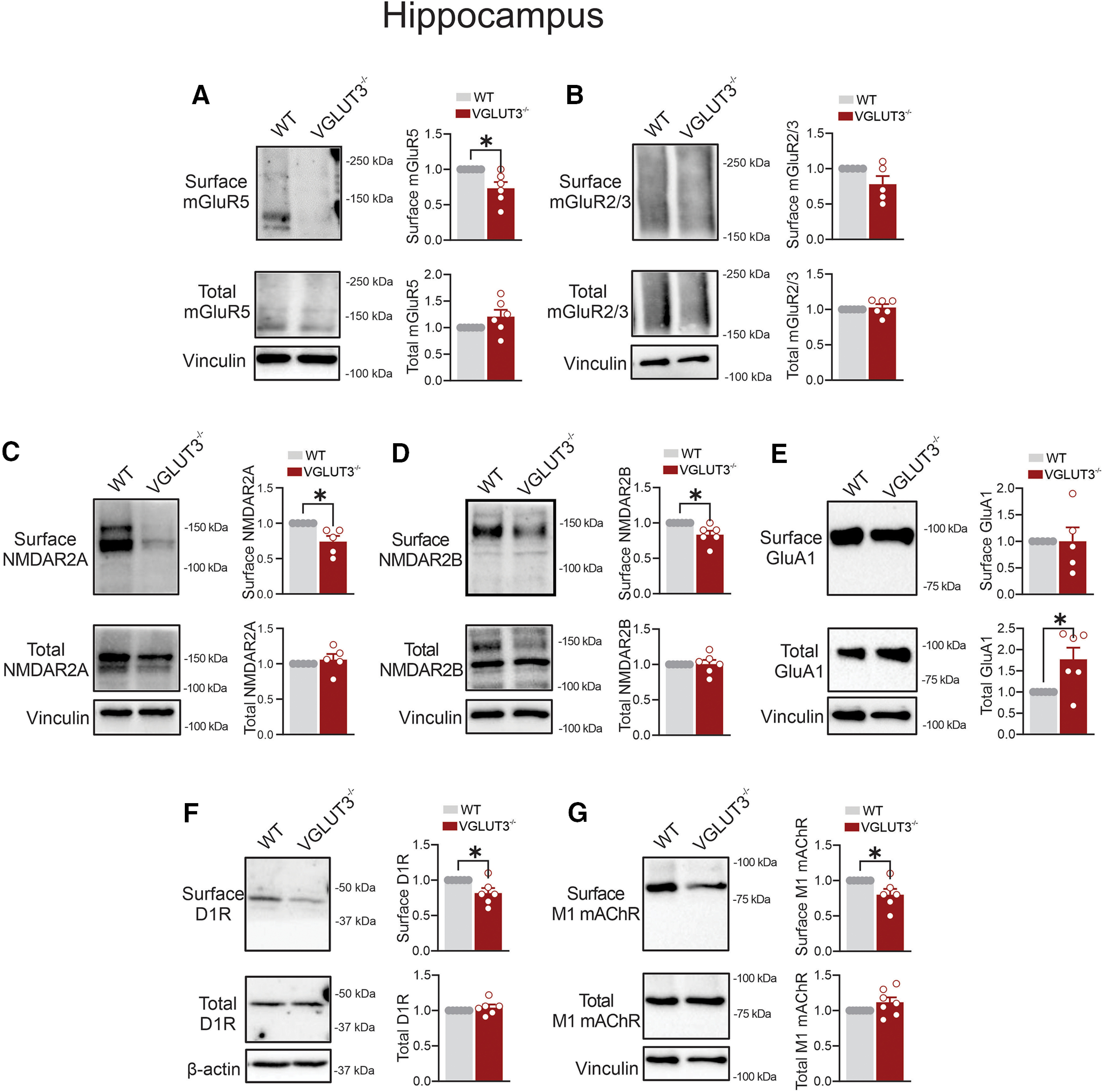
The effect of VGLUT3 deletion on glutamate and nonglutamate receptors expression in the mouse hippocampus. ***A–G***, Representative immunoblots and quantification of cell surface and total levels of mGluR5 (***A***), mGluR2/3 (***B***), NMDAR2A (***C***), NMDAR2B (***D***), GluA1 (***E***), D1R (***F***), and M1 mAChR (***G***) in hippocampal lysates from VGLUT3^−/−^ and C57BL/6 (WT) male mice. Data are the mean ± SEM (*n* = 5–6 mice/group), normalized to the respective surface or total WT values. *p < 0.05 assessed by unpaired Student’s *t* test.

Within the hippocampus, mGluRs and iGluRs are expressed in both excitatory glutamatergic and inhibitory GABAergic interneurons, with distinct patterns for each receptor subtype (Reiner and Levitz, 2018). Hippocampal VGLUT3 is found in subpopulations of GABAergic interneurons, cholecystokinin-positive (CCK^+^) basket cells, as well as in subsets of VGLUT3-expressing serotoninergic varicosities (Somogyi et al., 2004; Amilhon et al., 2010). In the hippocampus of VGLUT3^−/−^ mice, we found that cell surface mGluR5 levels were significantly reduced, with no change in their total expression levels (Fig. 2*A*). In addition, both cell surface and total expression levels of mGluR2/3 remained unaffected by VGLUT3 deletion in mice (Fig. 2*B*). However, cell surface fractions, but not total expression, of NMDAR2A and NMDAR2B were significantly reduced in the hippocampus of VGLUT3^−/−^ mice (Fig. 2*C*,*D*). Furthermore, total but not cell surface GluA1 levels were elevated in the hippocampus of VGLUT3^−/−^ mice (Fig. 2*E*). The loss of VGLUT3 reduced the cell surface levels of D1R and M1 mAChR without altering their total expression in the hippocampus (Fig. 2*F*,*G*).

### VGLUT3 loss increases the total expression of mGluR2/3, AMPARs, and cell surface mGluR5 levels in the striatum

**Figure 3.**
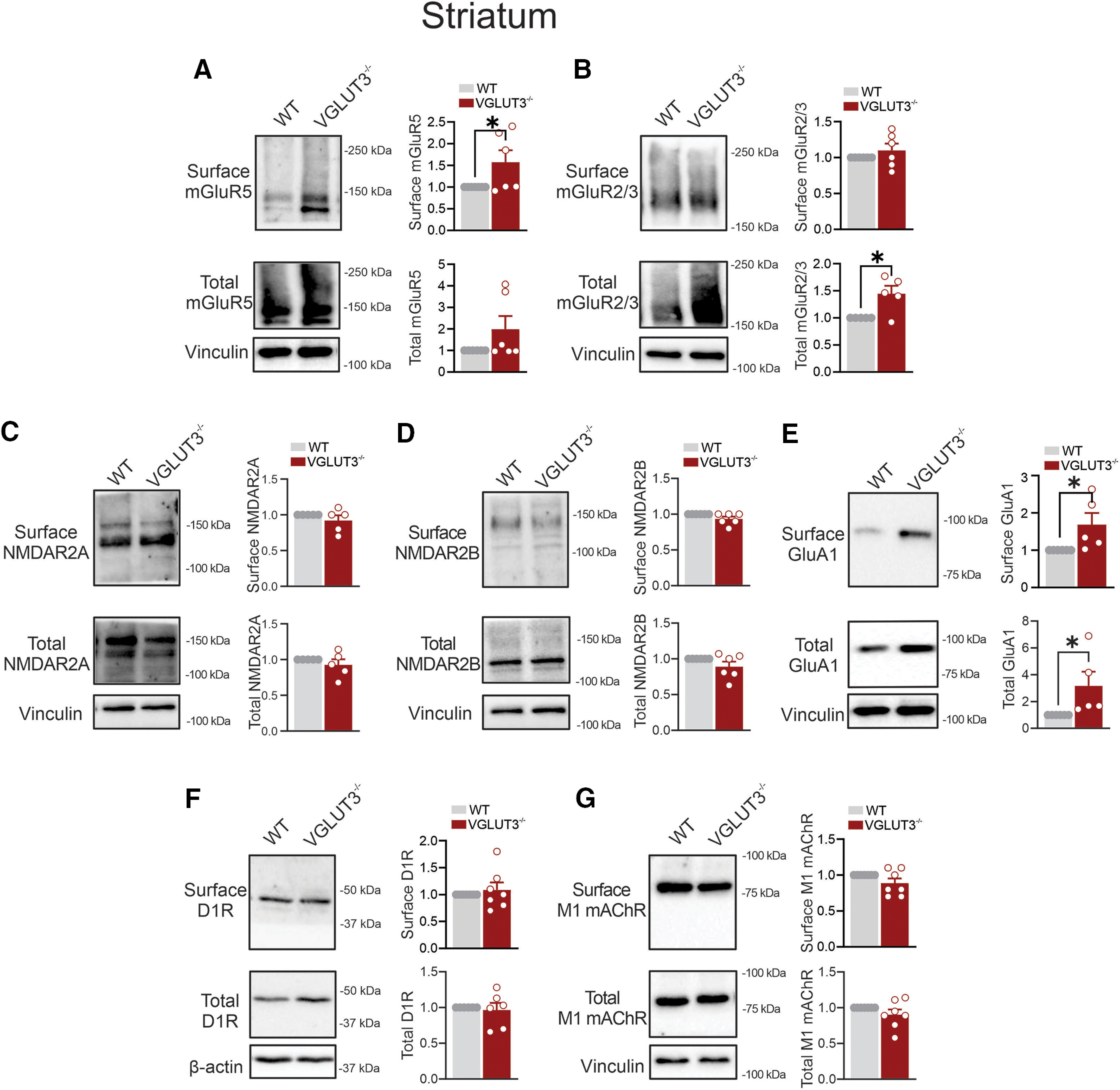
The effect of VGLUT3 deletion on glutamate and nonglutamate receptor expression in the mouse striatum. ***A–G***, Representative immunoblots and quantification of cell surface and total levels of mGluR5 (***A***), mGluR2/3 (***B***), NMDAR2A (***C***), NMDAR2B (***D***), GluA1 (***E***), D1R (***F***), and M1 mAChR (***G***) in striatal lysates from VGLUT3^−/−^ and C57BL/6 (WT) male mice. Data are the mean ± SEM (*n* = 5–6 mice/group), normalized to the respective surface or total WT values. *p < 0.05 assessed by unpaired Student’s *t* test.

Striatal VGLUT3 signaling is chiefly mediated via cholinergic interneurons (Higley et al., 2011; Gonzales and Smith, 2015). These interneurons use both acetylcholine and glutamate to fine-tune dopaminergic inputs onto the medium spiny neurons, ultimately regulating striatal network activities (Gras et al., 2008; Higley et al., 2011; Sakae et al., 2015). We found that the loss of VGLUT3 increased cell surface mGluR5 levels in the striatum, with no detectable changes in cell surface mGluR2/3 levels (Fig. 3*A*,*B*). However, total mGluR2/3, but not mGluR5, levels were significantly elevated in the striatum of VGLUT3^−/−^ mice (Fig. 3*A*,*B*). We also detected no changes in the total or cell surface expression of NMDAR2A and NMDAR2B in the striatum of VGLUT3^−/−^ mice compared with WT mice (Fig. 3*C*,*D*). Yet, VGLUT3 loss elevated both cell surface and total expression levels of GluA1 subunits (Fig. 3*E*). The expression of D1R and M1 mAChR in the striatum remained unaffected by VGLUT3 deletion (Fig. 3*F*,*G*).

## Discussion

Despite the robust evidence supporting the role of VGLUT3 in regulating nonglutamatergic neurotransmission (for review, see Trudeau and El Mestikawy, 2018; Ibrahim et al., 2020), little is known about how VGLUT3-associated networks regulate glutamatergic neurotransmission. As summarized in Table 1, we show that genetic deletion of VGLUT3 differentially alters the expression of glutamate and nonglutamate receptors in several brain areas. Specifically, VGLUT3 ablation upregulates the expression of mGluR5 and mGluR2/3, and reduces cell surface NMDA2B and D1R levels in the cerebral cortex. In the hippocampus, VGLUT3 loss reduces cell surface levels of mGluR5, NMDAR2A/2B, as well as D1Rs and M1 mAChRs. Cell surface mGluR5 and total mGluR2/3 densities are increased in the striatum of VGLUT3^−/−^ mice. Last, the loss of VGLUT3 upregulates AMPAR subunit GluA1 in the cortex, the hippocampus, and the striatum. Together, these findings complement and provide further evidence that VGLUT3, despite its low abundance, is a key modulator of GluR densities across the brain, highlighting its role in glutamatergic transmission, synaptic plasticity, and brain circuit homeostasis.

**Table 1:** Summary of changes in cell surface and total levels of receptors in the different brain regions of VGLUT3^–/–^ mice

Brain regions	mGluR5		mGluR2/3		NMDAR2A		NMDAR2B		GluA1		D1R		M1 mAChR	
	Surface	Total	Surface	Total	Surface	Total	Surface	Total	Surface	Total	Surface	Total	Surface	Total
Cerebral cortex	↑	↑	-	↑	-	-	↓	-	↑	↑	↓	-	-	-
Hippocampus	↓	-	-	-	↓	-	↓	-	-	↑	↓	-	↓	-
Striatum	↑	-	-	↑	-	-	-	-	↑	↑	-	-	-	-

(-) denotes no change.

Evidence indicates that VGLUT3 modulates cortical and hippocampal microcircuits via CCK^+^ basket cells (Herzog et al., 2004; Somogyi et al., 2004; Nguyen et al., 2020). These interneurons use VGLUT3-dependent glutamate to dampen GABAergic currents onto pyramidal neurons via feedback activation of presynaptic mGluR autoreceptors (Fasano et al., 2017). VGLUT3 transmission also mediates excitatory inputs onto pyramidal neurons as well as interneurons via activation of AMPARs and NMDARs (Nguyen et al., 2020). Moreover, VGLUT3-expressing basket cells form synaptic contacts with mGluR5 on pyramidal neurons in the cortical and cortex-like amygdaloid regions (Omiya et al., 2015). Consistent with these reports, we observed upregulation in both mGluR5 and AMPAR subunit GluA1 in the cortex upon deletion of VGLUT3. Yet, cell surface levels of NMDAR2B, but not NMDAR2A, were decreased in the cortex of VGLUT3^–/–^ mice. This intriguing observation suggests that VGLUT3 exerts sophisticated regulation of some NMDAR subpopulations in cortical neurons. Interestingly, NMDAR2B-containing receptors exhibit slower deactivation kinetics and facilitated surface mobility compared with NMDAR2A-containing receptors (Vicini et al., 1998; Groc et al., 2006), ultimately affecting the nature of the plasticity response (Paoletti et al., 2013). Thus, the reduction in NMDAR2B cell surface levels in VGLUT3^−/−^ cortex suggests that VGLUT3 may alter the induction thresholds of long-term potentiation (LTP) and long-term depression (LTD) in the cortical neurons. This agrees with the observed decreases in cell surface D1R levels and upregulation in mGluR2/3 levels in VGLUT3^−/−^ cortex: both actively modulate LTP and LTD induction/maintenance in the cortex (Otani et al., 1999; Huang et al., 2004; Joffe et al., 2020, 2021). Together, our findings further support the role of VGLUT3 in modulating the balance between LTP and LTD plasticity responses in corticohippocampal networks (Fasano et al., 2017).

The observed reductions in mGluR5 and NMDAR membrane recruitment in VGLUT3^−/−^ hippocampus suggest an adaptive reduction in glutamatergic transmission strength in the hippocampal circuits. Both group I mGluRs and NMDARs represent key players in regulating synaptic metaplasticity in hippocampal neurons (Reiner and Levitz, 2018). Similarly, inputs from VGLUT3^+^ interneurons are reported to modulate metaplasticity in glutamatergic synapses within the regional circuits of the hippocampus (del Pino et al., 2017; Fasano et al., 2017). Therefore, It is possible that the reduction in mGluR5 and NMDAR cell surface levels may underlie altered metaplasticity in the ventral hippocampus of VGLUT3^−/−^ mice (Fasano et al., 2017). We also noted a decrease in cell surface densities of D1R and M1 mAChR, both of which are expressed in VGLUT3^+^ interneurons and regulate synaptic plasticity mechanisms and memory-processing functions in the hippocampus (Cea-del Rio et al., 2010; Furini et al., 2014; Dennis et al., 2016; Puighermanal et al., 2017). Collectively, these changes in glutamatergic and nonglutamatergic receptor topographies might explain the impaired working memory and cognitive flexibility previously reported in VGLUT3^−/−^ mice (Fazekas et al., 2019).

VGLUT3 signaling plays an important role in striatal microcircuits involved in reward-guided behaviors (Ibrahim et al., 2020). We show here that VGLUT3 loss triggers an increase in cell surface mGluR5, as well total mGluR2/3 levels in the striatum. Both receptors play a well characterized role in the striatal circuits modulating drug reward and motor function (Kenny and Markou, 2004; Conn et al., 2005; Paterson and Markou, 2005; Peters and Kalivas, 2006). Thus, our findings indicate that VGLUT3, via its dynamic regulation of striatal mGluR densities, can play a critical role in the neuropathophysiology of drug addiction and movement disorders (Sakae et al., 2015; Areal et al., 2019; Ibrahim et al., 2020).

An intriguing aspect of our findings is the persistent upregulation of AMPAR subunit GluA1 across cortical, hippocampal, and striatal regions of VGLUT3^–/–^ mice. This suggests that AMPAR activation might be regulated by VGLUT3 neurotransmission, independent of NMDARs. Previous reports have shown that VGLUT3-expressing interneurons mediate excitatory inputs via AMPAR activation in the prefrontal cortex (Nguyen et al., 2020), the hippocampus (Pelkey et al., 2020), and the striatum (Nelson et al., 2014). Furthermore, synaptic scaling of AMPARs is directly implicated in central neural circuits that control spatial working memory functions (Bannerman et al., 2004; van Vugt et al., 2020) and reward behavior (Sutton et al., 2003). Thus, the observed AMPAR alterations could potentially be implicated in the impaired working memory and exacerbated cocaine hedonic effects reported in VGLUT3^−/−^ mice (Sakae et al., 2015; Fazekas et al., 2019) which warrants further investigation.

It is worth noting that our findings are subject to at least two limitations. First, while cell surface biotinylation is efficient in yielding highly purified plasma membrane proteins, the technique cannot provide spatial information on precise distribution of the receptors in different synaptic domains. Second, we focused on male VGLUT3^−/−^ mice as prior studies did not report major differences between male and female knock-out mice, in either their phenotype or network activities (Amilhon et al., 2010; Higley et al., 2011; Divito et al., 2015; Ramet et al., 2017). Nonetheless, we cannot rule out sex-dependent changes in GluR densities in VGLUT3^−/−^ mice.

In summary, we provide evidence that VGLUT3, despite its low abundance, can be a key regulator of mGluR and iGluR densities in different brain regions, highlighting the role of VGLUT3 in synaptic function and plasticity. Moreover, because VGLUT3 regulates specialized brain circuitries involved in reward processing, emotional states, and motor coordination, this neuronal adaption of GluR densities might provide a more comprehensive understanding of VGLUT3-dependent mechanisms underlying various neurologic and neuropsychiatric disorders such as anxiety (Amilhon et al., 2010), drug addiction (Sakae et al., 2015), or motor disorders such as Parkinson’s disease (Divito et al., 2015; Ribeiro et al., 2017).

## Synthesis

Reviewing Editor: Bong-Kiun Kaang, Seoul National University Decisions are customarily a result of the Reviewing Editor and the peer reviewers coming together and discussing their recommendations until a consensus is reached. When revisions are invited, a fact-based synthesis statement explaining their decision and outlining what is needed to prepare a revision will be listed below. The following reviewer(s) agreed to reveal their identity: NONE.

The authors report the genetic ablation study of type 3 vesicular glutamate transporter (VGLUT3) which is an atypical glutamate transporter but is vital for regulation of glutamate

signaling in various brain microcircuits. It is well written but needs some minor revisions listed below.

Reviewer 1:

VGLUT3 is a critical player in glutamate signaling through modulating glutamate release. In this manuscript, the authors investigated the effect of VGLUT3 KO on the expression of glutamate receptors, dopamine D1 receptors, and M1 mAchR across different brain regions using VGLUT3 constitutive knockout mice. They found that, in the

cerebral cortex, VGLUT3 deletion upregulated the expression of mGluR5 (total and membrane), mGluR2/3 (total alone) and GluA1 (total alone) while the membrane expression of NR2B and D1 is downregulated. In the hippocampus, the membrane expression of mGlR5, NR2A, NR2B, D1, and M1 is decreased while GluA1 total expression is increased. In the striatum, the surface expression of mGluR5m GluA1 and the total expression of GluA1 are increased.

These findings not only provided evidence for an important role of VGLUT3 in modulating glutamatergic neurotransmission, but they can also be helpful references for scientists working in the neurotransmitter receptor research area. The manuscript was well structured, written clearly, and the project is very straightforward. I do not have many concerns over the results; however, I have a couple of suggestions for authors.

1. To measure the membrane expression of the receptor in tissue, it might be better to use membrane fractionation directly. There are several commercially available kits, and they work very well and are easy to use.

2. To present the membrane expression of a protein, it should include a membrane marker such as TfR.

3. The authors may want to add their speculation/hypothesis regarding the observed differences in the receptor expression among the three receptor families. It would be great if they can tie these changes with physiological/pathological conditions.

Reviewer 2:

Using VGLUT3, atypical vesicular glutamate transporter, knockout in mice, the authors showed that VGLUT3 differentially regulates glutamate receptor densities in various brain regions. Interestingly enough, they found that despite the limited abundance of VGLUT3, its genetic deletion differentially alters the expression of glutamate and non-glutamate receptors in several brain areas. The data interpretation is sound and the manuscript is well written. I have only a minor point to improve the manuscript.

1. They found that VGLUT3 ablation upregulates the

expression of some types of mGluRs and AMPARs while reducing cell surface NMDARs, D1R and M1R levels in some brain areas. In Discussion, the authors argue that GluN2A containing receptors are required for LTP, while GluN2B containing receptors are critically important for LTD. This notion may be true, but still controversial as some other reports demonstrated this simple dichotomy may not always apply at least depending on brain regions (Zhao et al, 2005).

Zhao MG et al., Roles of NMDA NR2B subtype receptor in prefrontal long-term potentiation and contextual fear memory. Neuron. 2005 Sep 15;47(6):859-72.

GluN2A also showed some reduction in the hippocampus of VGLUT3 KO, despite less reduction than GluN2B. This discussion part needs to be revised carefully.
